# Isolated Uterine Metastasis of Invasive Ductal Carcinoma

**DOI:** 10.1155/2013/793418

**Published:** 2013-03-14

**Authors:** Deniz Arslan, Deniz Tural, Ali Murat Tatlı, Emre Akar, Mükremin Uysal, Gülgün Erdoğan

**Affiliations:** ^1^Department of Internal Medicine, Division of Medical Oncology, Medical Faculty, Akdeniz University, Antalya, Turkey; ^2^Department of Internal Medicine, Division of Medical Oncology, Cerrahpasa Medical Faculty, Istanbul University, 34098 Istanbul, Turkey; ^3^Cerrahpasa Medical Faculty, Istanbul University, 34098 Istanbul, Turkey; ^4^Department of Pathology, Medical Faculty, Akdeniz University, Antalya, Turkey

## Abstract

*Introduction*. Most common metastasis sites of breast cancer are the lungs, bones, liver, and brain, whereas uterine involvement by metastatic breast disease is rare. Metastatic carcinoma of the uterus usually originates from other genital sites, most commonly being from the ovaries. Invasive lobular carcinoma spreads to gynecologic organs more frequently than invasive ductal carcinoma. *Case Report*. A 57-year-old postmenopausal woman was diagnosed with breast carcinoma 2 years ago and modified radical mastectomy was performed. Pathological examination of tumor revealed invasive ductal carcinoma, stage IIIc. She presented with abdominal pain and distension. Diagnostic workup and gynecologic examination revealed lesions that caused diffuse thickening of the uterus wall. Endometrial sampling was performed for confirmation of the diagnosis. She underwent total abdominal hysterectomy and bilateral salpingo-oophorectomy. Breast carcinoma metastases in endometrium and myometrium were confirmed histopathologically and immunohistochemically. *Conclusion*. We herein report the first case of isolated uterine patient who had invasive ductal carcinoma of breast.

## 1. Introduction

Most common metastasis sites of breast cancer are the lungs, bones, liver, and brain, whereas uterine involvement by metastatic breast disease is rare. Metastatic carcinoma of the uterus usually originates from other genital sites, most commonly being from the ovaries [1, 2]. Invasive lobular carcinoma spreads to gynecologic organs more frequently than invasive ductal carcinoma [3]. And if the uterus would be infiltrated, abnormal uterine bleeding is usually the most common manifestation [4]. However, most of the uterine metastases are found on autopsy [5].

Anastrozole is the third-generation aromatase inhibitor that reduces serum estrogen level and affects through competitive inhibition of enzyme aromatase. In contrast to tamoxifen, endometrium should be expected to be safer under anastrozole treatment. With all this information, risk of uterine metastasis is thought to be lower in concepts of invasive ductal carcinoma and anastrozole therapy. 

We herein report the first case of isolated uterine metastasis under anastrozole treatment in a postmenopausal patient who had invasive ductal carcinoma of breast.

## 2. Case Report

**Figure 1:**
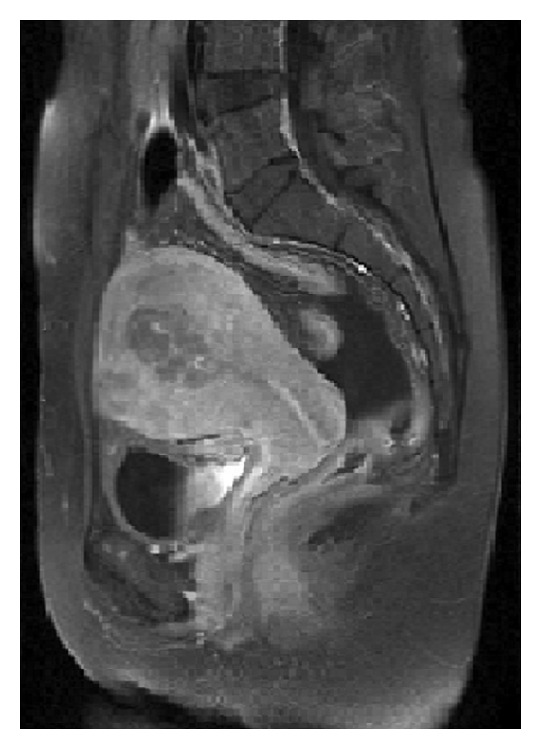
Pelvic MRI. Increase in diffuse thickness of uterus.

**Figure 2:**
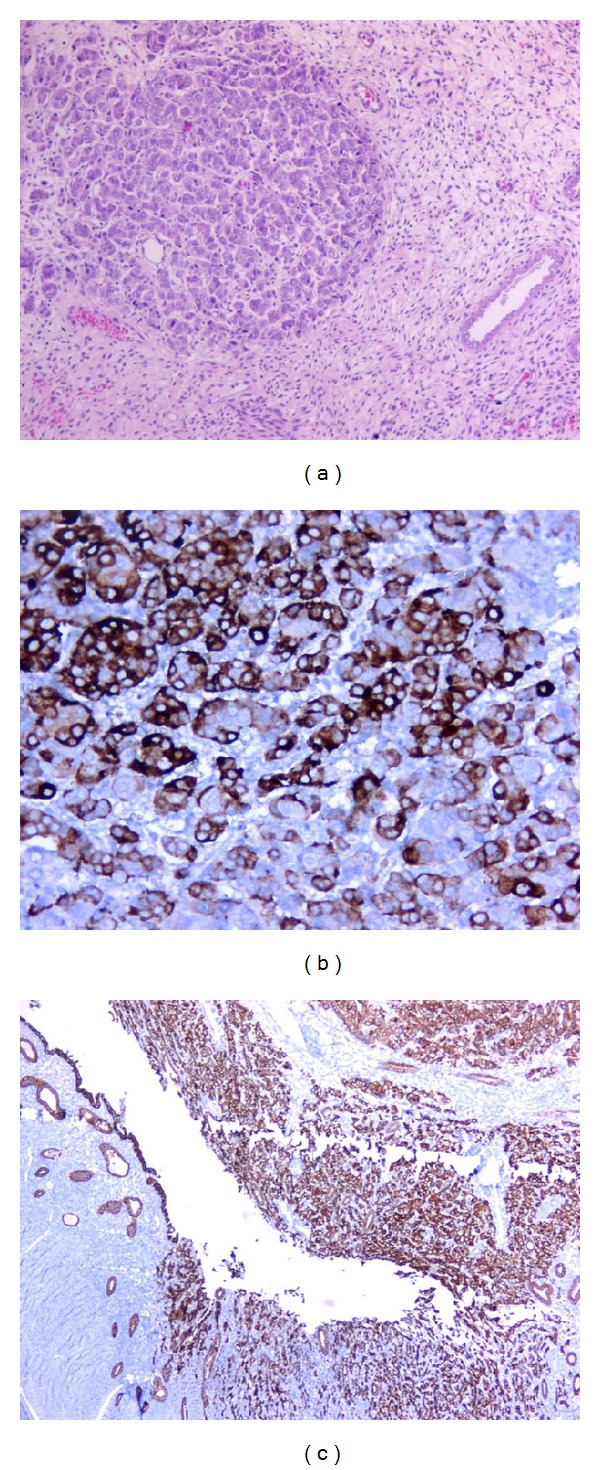
Pathological examination. Pathological microscopic examination demonstrating malign epithelial tumor cells ((a); hematoxylin-eosin, ×100), immunohistochemical staining positive for GCDFP-15 ((b); ×200), and pancytokeratin ((c); ×40).

A 57-year-old postmenopausal woman was diagnosed with breast carcinoma 2 years ago and modified radical mastectomy was performed. Pathological examination of tumor revealed invasive ductal carcinoma, stage IIIc (T1b, N3a, M0, and G2). Immunohistochemical staining showed strongly positive for estrogen receptors (ER) and progesterone receptors (PR), negative for cerbB2. She received adjuvant chemotherapy with 3 cycles of cyclophosphamide, doxorubicin, and 5-fluorouracil followed by 3 cycles of docetaxel. Radiotherapy of 5000 cGy was also performed to the chest wall and right supraclavicular and right axillary areas. 5 years of anastrozole treatment was planned. However, during the 16th month of endocrine therapy, she presented with abdominal pain and distension. Diagnostic workup and gynecologic examination revealed lesions that caused diffuse thickening of the uterus wall (Figure 1). Endometrial sampling was performed for confirmation of the diagnosis. She underwent total abdominal hysterectomy and bilateral salpingo-oophorectomy. Breast carcinoma metastases in endometrium and myometrium were confirmed histopathologically and immunohistochemically with positivity of pancytokeratin and gross cystic disease fluid protein-15 (GCDFP-15) and negativity of Melan A, CD 10, caldesmon, and alpha-fetoprotein (AFP) (Figure 2). She is under the exemestane treatment that was commenced after the surgery. 

## 3. Discussion

Metastases to the female genital tract from extragenital cancers are rare. Breasts and the gastrointestinal tract are the most common sites of the primary tumor. Ovaries are most frequently affected by metastases accounting for 75.8%, followed by vagina (13.4%), uterine corpus (4.7%), cervix (3,4%), vulva (2%), and salpinx (0.7%) [1]. Uterine metastases usually occur secondary to local lymphatic spread from the ovarian involvement and thus isolated uterine metastases from the extragenital tumors are rare and probably hematogenous [4].

 Anatomic distribution of metastases in the uterine corpus was investigated, and it was demonstrated that involvement of the myometrium only accounts for 63.5%, followed by myometrium and endometrium (32.7%) and endometrium only (3.8%) [2]. Initial symptoms of the uterine metastasis depend on the anatomic involvement site. Abnormal uterine bleeding is often the first symptom when the endometrium is involved. However, if the infiltration would affect myometrium only, patients may often be asymptomatic [4]. In our case, the patient presented with abdominal pain and distension. Hence, it should be considered that initial symptoms may fail to show the site of disease. With the aim of detecting metastatic disease early, any symptom should be thought about gynecologic spread, and routine follow-up gynecologic examinations should be performed in asymptomatic patients.

Histologic types of breast cancer are invasive ductal carcinoma (IDC) and invasive lobular carcinoma. IDC accounts for approximately 70–75% of all breast cancers compared to ILC that only accounts for 5–20%. Despite of its lower incidence in all breast cancers, ILC is the most frequent histologic type that metastasizes to the female genital tract in more than 80% of all cases [6]. Nevertheless, it should be kept in mind that IDC also may metastasize to the gynecologic organs alone, as seen in our case.

Anastrozole has been shown to be superior to tamoxifen in cancer treatment. In consideration of relationship between endometrium pathologies and tamoxifen, anastrozole is expected to be safer and more effective in treatment of breast cancer and in prevention of recurrences and metastases [7, 8]. Even in the literature, there are some studies that reported successful use of anastrozole in the treatment of endometrial hyperplasia and carcinoma [9, 10]. But interestingly, as seen in our case, uterine metastases may grow in patients receiving anastrozole therapy. A few number of reports regarding gynecologic metastases from breast cancer under anastrozole treatment were published [6, 11]. When the interesting feature of isolated uterine metastasis under anastrozole therapy was taken, comments can be made according to histologic type of primary breast lesion. For that reason, ILC has been reported as histologic type in the literature. The condition of uterine metastasis of IDC was also reported. But none of these patients have been under anastrozole treatment [4, 12, 13]. 

This is the first report of a patient receiving anastrozole and primary histologic type of IDC metastasized to the uterus only. In consideration of this study's limitations, due to reporting only one patient and its very rare features, this study cannot prove any correlation between anastrozole treatment and uterine metastases. But physicians should be aware and concerned about isolated uterine metastases that may grow in the patients receiving anastrozole. Routine gynecological follow-up examination should be carried out in breast cancer patients under anastrozole therapy as well as tamoxifen. Additionally, it is important to distinguish the uterine lesions whether primary or metastatic because of the different treatment options.
